# Layer‐Dependent Phonon Polaritons in hBN Resolved by Photo‐Induced Force Spectroscopy

**DOI:** 10.1002/advs.76087

**Published:** 2026-06-12

**Authors:** Amin Hajarian, Jiwoo Seo, SungWoo Nam

**Affiliations:** ^1^ Department of Mechanical and Aerospace Engineering University of California Irvine California USA; ^2^ Department of Materials Science and Engineering University of California Irvine California USA

**Keywords:** hexagonal boron nitride, layer dependence, phonon polaritons, photo‐induced force microscopy, resonance shift

## Abstract

Hexagonal boron nitride (hBN) is a widely studied van der Waals material that supports highly confined phonon polaritons—hybrid light‐matter quasiparticles arising from the coupling of infrared photons with optical phonons. While prior works—primarily using scattering‐type near‐field optical microscopy (s‐SNOM)—have explored the effect of hBN layer number on the wavelength and propagation characteristics of phonon polaritons, the direct spectroscopic investigation of their resonance energy as a function of layer number has remained largely unaddressed. In this study, we present the first systematic spectroscopic analysis of layer‐dependent phonon polariton resonance frequencies in hBN, using photo‐induced force microscopy (PiFM). By probing hBN with layer numbers ranging from approximately 10 to 60, we uncover clear and opposing trends in the resonance behavior of the two principal Reststrahlen bands. Specifically, we observe that the in‐plane phonon polariton resonance exhibits a blue shift with increasing layer number, while the out‐of‐plane resonance shows a red shift. Additionally, the peak intensity in the out‐of‐plane band increases with layer number, whereas the in‐plane resonance becomes weaker and broader. These findings reveal critical insights into the optical response of hBN across different layer regimes and underscore the importance of layer number as a tuning parameter in nanophotonic applications.

## Graphical abstract

Layer‐dependent phonon polariton resonances in hexagonal boron nitride are systematically investigated using photo‐induced force microscopy. The in‐plane resonance exhibits a blueshift, while the out‐of‐plane resonance shows a redshift with increasing thickness. These results establish layer number as a key parameter for tuning polaritonic responses in van der Waals materials for nanophotonic applications.

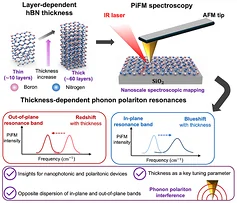

## 1 Introduction

Phonon polaritons represent a paradigmatic class of hybrid light‐matter quasiparticles that emerge from the strong coupling between infrared photons and optical phonons within polar dielectric materials, fundamentally transforming nanoscale electromagnetic phenomena [1, 2, 3, 4]. These exotic states exist within the Reststrahlen spectral region, where the material's permittivity exhibits opposite signs, enabling highly confined electromagnetic modes with wavelengths orders of magnitude smaller than their free‐space counterparts. The significance of phonon polaritons in nano‐photonics stems from their unique ability to circumvent the diffraction limit while maintaining exceptionally low dissipative losses, a combination elusive in plasmonic systems [5, 6, 7, 8].

Among van der Waals materials, hexagonal boron nitride (hBN) has emerged as a premier platform for hosting hyperbolic phonon polaritons, where anisotropic optical properties enable unprecedented directional control and extreme field confinement down to interatomic scales [9, 10, 11, 12, 13]. This extraordinary light‐matter interaction regime has catalyzed transformative advances across diverse nanophotonic applications, including super‐resolution imaging [14], surface‐enhanced vibrational spectroscopy [15], sub‐diffraction waveguiding [16], and directional thermal management [17, 18]. The atomically precise tunability of polariton properties through thickness control positions phonon polaritons as cornerstone elements in quantum nano‐photonics and next‐generation opto‐electronic devices.

Building on this foundation, the atomic‐layer precision achievable in van der Waals materials through layer‐by‐layer assembly (i.e., mechanical exfoliation and stacking) presents an unprecedented opportunity to engineer phonon polariton properties with layer‐dependent tunability. Similar to the tunability seen in graphene plasmons via selective doping and structural modulation [19, 20, 21], phonon polaritons in hBN can be finely adjusted by manipulating intrinsic material parameters, particularly the number of atomic layers. This precise modulation at the single‐layer level systematically influences phononic confinement effects, dispersion characteristics, and resonance behaviors, enabling a level of optical control fundamentally inaccessible in conventional bulk materials. Understanding these layer‐dependent interactions provides crucial insights into reduced‐dimensional light‐matter coupling and opens avenues for designing innovative polaritonic devices.

Although the potential of layer number‐controlled hBN for polaritonic applications is well recognized, prior studies have largely focused on isolated phenomena, such as variations in oscillation strength, polariton wavelengths, and thermal conductivity with thickness [22, 23]. Notable contributions include the work of Jia et al. [24], who showed significant modulation of plasmon‐phonon hybridization strength through layer‐by‐layer thickness control, and investigations by Dai et al. [25, 26] and Shi et al. [27], who reported systematic evolutions in polariton dispersion and wavelength scaling. However, these studies have primarily focused on isolated aspects of thickness dependence, lacking comprehensive spectroscopic analysis that simultaneously addresses mode‐specific frequency shifts, intensity variations, and dispersion characteristics across a systematic range of layer numbers. The absence of detailed investigations in the intermediate thickness regime (< 100 layers of hBN) represents a significant knowledge gap that limits both fundamental understanding and practical implementation of thickness‐engineered hBN devices.

To systematically explore layer‐dependent properties of phonon polaritons in hBN, employing high‐spectral‐resolution spectroscopic techniques is crucial [28]. While scattering‐type scanning near‐field optical microscopy (s‐SNOM) has been extensively used [29], it primarily provides spatial interference patterns and amplitude information [14, 27], facing limitations in direct spectral measurement due to complexity and background noise [30, 31]. In contrast, photo‐induced force microscopy (PiFM) combines high spatial resolution with direct spectroscopic capabilities, significantly reducing background contributions. PiFM detects photo‐induced forces at the AFM tip rather than scattered light, which means the contrast comes from local tip–sample interactions instead of far‐field background signals. This inherently suppresses far‐field scattering noise that s‐SNOM often suffers from [32, 33, 34]. PiFM uniquely allows effective mapping of modes in spectral regions, such as the lower Reststrahlen band, where s‐SNOM faces challenges due to its reliance on interferometric detection and the more limited availability of suitable continuous‐wave laser sources [35]. Despite these advantages, PiFM has only been utilized, demonstrating basic detection capabilities, such as the observation of phonon‐polariton edge‐induced interference fringes or qualitative near‐field contrast imaging for hBN.

In this work, we present a spectroscopic study of layer‐dependent phonon polariton behaviors in hexagonal boron nitride using PiFM. By examining hBN samples in the critical intermediate thickness range of ∼10–60 layers—a regime less thoroughly investigated in previous studies—we establish a quantitative correlation between resonance energies and layer number across both Reststrahlen bands. Our analysis reveals distinct scaling behaviors in the in‐plane and out‐of‐plane bands, demonstrating layer number as a parameter for engineering hBN's optical response. Unlike most works that focus primarily on the in‐plane band, we perform comprehensive PiFM imaging and spectroscopic characterization across both bands. We further contribute to the spectroscopic characterization of the relationship between layer number and polaritonic resonances beyond phonon polariton imaging. These findings provide design parameters for nanophotonic applications, enabling tailored optical properties through controlled layer modulation.

## 2 Results and Discussion

### 2.1 Layer‐Dependent Polariton Wavelength

To investigate the layer‐dependent properties of phonon polaritons in hBN, we prepared multiple hBN samples with layer number ranging from approximately 10–60 layers (∼ 3–20 nm) through mechanical exfoliation. The fabrication process began with mechanical exfoliation of bulk hBN, followed by transfer onto polydimethylsiloxane (PDMS) gel‐pak substrates. The exfoliated flakes were inspected under optical microscopy to identify samples of desired thickness before careful transfer onto 300 nm SiO_2_/Si substrates using a transfer stage operated under controlled low oxygen and humidity conditions through argon and nitrogen purging.

To avoid substrate‐induced artifacts in our measurements, we focused on hBN flakes with thicknesses ranging from 10 to 60 layers (approximately 3–20 nm). Prior studies have shown that ultrathin hBN layers in direct contact with SiO_2_/Si substrates are prone to substrate‐induced strain, which can shift the phonon frequencies observed in Raman spectroscopy [36]. This strain effect compromises the spectral stability needed for accurate phonon polariton resonance analysis. By selecting moderately thick flakes, we minimized substrate interactions and ensured that the intrinsic phononic properties of hBN dominated the measured response (Figure S1).

**FIGURE 1:**
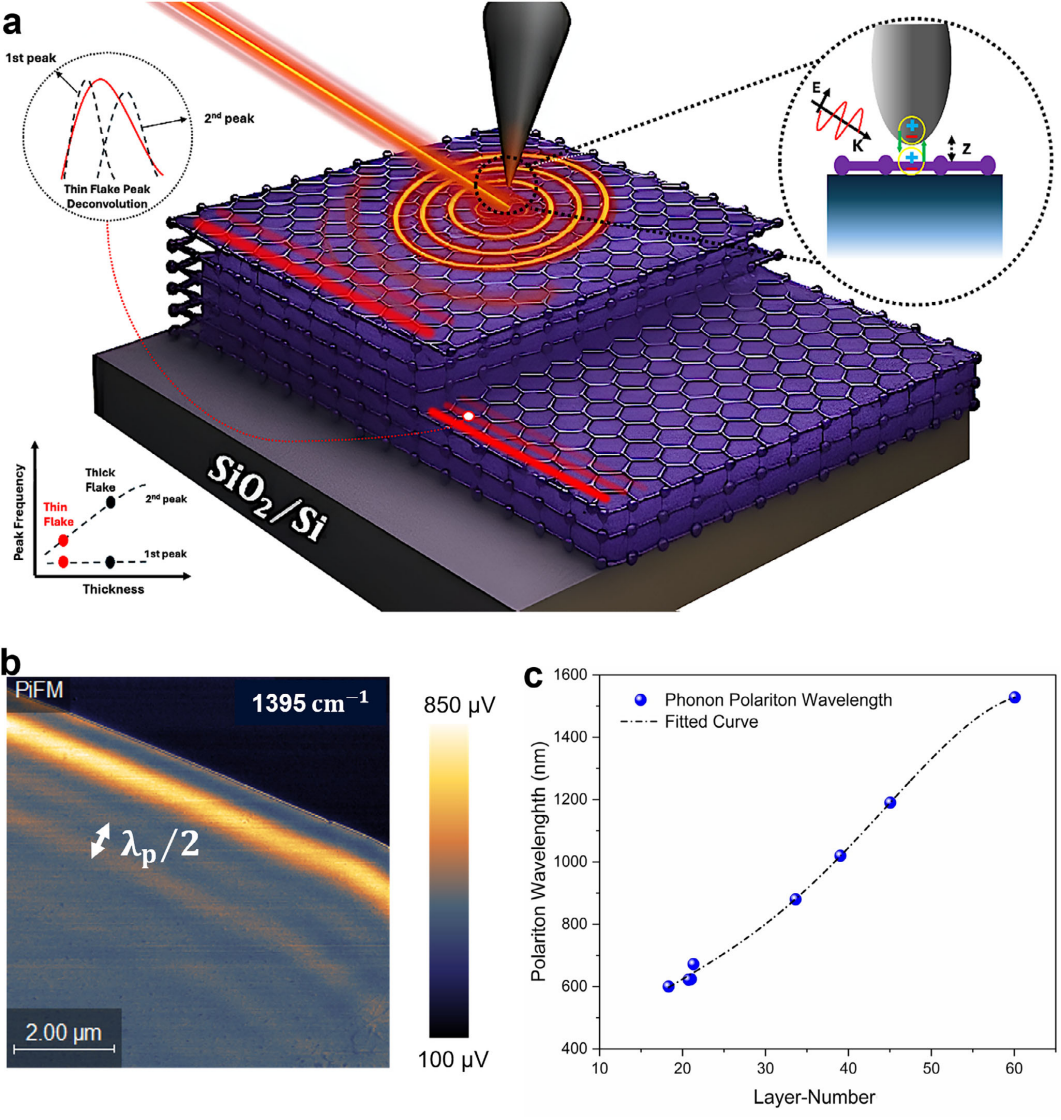
Phonon polariton wavelength vs. thickness. (a) Schematic of PiFM on an hBN with varying thicknesses. (b) PiFM images showing interference fringes near the edge of the hBN sample with a thickness of 27 nm. (c) Phonon polariton wavelength variation as a function of hBN layer number.

Figure 1a illustrates the schematic of our PiFM experimental setup probing an hBN flake with varying layer thickness. The PiFM system was equipped with Au‐coated metallic tips operating at dual resonance frequencies: the second resonance frequency (w_2_≅ 1600 kHz) with stiffness of 1479 N/m for high‐resolution topography measurements, and the first resonance frequency (w_1_≅ 250 kHz) for detecting photo‐induced forces with enhanced sensitivity. The system was coupled with a tunable quantum cascade laser (QCL) providing wavenumber sweeps across 770–1900 cm^−1^, encompassing the spectral range relevant to hBN phonon polaritons. The laser modulation frequency (w_m_) was synchronized with the cantilever resonance frequencies using sideband detection mode (w_m_ = w_2_ ± w_1_) to maximize surface sensitivity while maintaining stable tip‐sample distances through careful control of drive amplitude and setpoint.

Figure 1b shows a representative PiFM image revealing distinct interference fringes localized near the hBN flake edge (see Figure S2). The interference fringes are clearly visible as periodic intensity modulations extending from the flake edge into the hBN region, with the fringe visibility and spacing providing quantitative information about the underlying polariton wavelength. These standing wave patterns emerge from the reflection of propagating phonon polaritons at the sample boundary, creating characteristic fringe structures that serve as direct signatures of polaritonic wave confinement.

The complex fringe structure from our PiFM imaging reveals the coexistence of two distinct phonon polariton components: tip‐launched roundtrip polaritons and direct edge‐launched polaritons. The tip‐launched roundtrip component originates from the AFM tip, which launches polaritons that propagate as circular waves to the edge of the flake, where they are reflected back to the tip, creating interference fringes with periodicity of half the phonon polariton wavelength (λ_p_/2). In contrast, the edge‐launched component has periodicity equal to the full polariton wavelength (λ_p_) and originates from direct scattering at the sharp edges of the sample [35]. This dual‐component interference pattern enables the determination of the complete polariton dispersion characteristics through geometric analysis of the standing wave structure. Our use of p‐polarized laser illumination enhances the visibility of tip‐launched (edge‐reflected) polaritons, as the strong vertical electric field component couples efficiently with the metallic AFM tip. This polarization configuration aligns with established literature showing that p‐polarized excitation favors tip‐launched modes, while s‐polarized illumination predominantly excites direct edge‐launched polaritons with reduced tip coupling efficiency [37].

Figure 1c shows the variation in phonon polariton wavelength as a function of hBN layer number for the in‐plane Reststrahlen band ($∼1360-1620\;cm^{-1}$), revealing a clear positive correlation between hBN layer number and polariton wavelength. The experimental data points, extracted from multiple samples across the intermediate layer numbers (10–60 layers), show systematic wavelength scaling. Consistent with prior works, we observe that as the hBN thickness increases, the distance between fringes also increases, indicating longer polariton wavelengths (see Figures S3–S5 for frequency‐dependent fringe evolution).

This layer number‐dependent wavelength scaling arises from the electromagnetic confinement effects in hBN's in‐plane hyperbolic band (type II). For the in‐plane Reststrahlen band, decreasing layer number leads to enhanced electromagnetic field confinement, resulting in larger wave vectors and consequently shorter wavelengths in thinner samples. As the hBN layer becomes thicker, the confinement is reduced, allowing for longer wavelength modes to be supported.

The PiFM technique uniquely enables visualization of polaritonic interference patterns with nanoscale spatial resolution while simultaneously providing spectroscopic information. While polaritonic interference fringes have been predominantly demonstrated in relatively thick hBN samples in previous literature [2, 12], the present study successfully images these patterns in thinner flakes where fringe visibility is typically reduced due to higher propagation losses and increased substrate coupling effects [25, 38].

### 2.2 Spectral Evolution of Edge‐Reflected Fringes and Multipeak Response

To further investigate how interference from the flake boundary modifies the optical response of hBN phonon polaritons, we performed systematic PiFM imaging and point‐spectroscopy across the edge‐reflected fringe pattern formed within a region of constant thickness.

**FIGURE 2:**
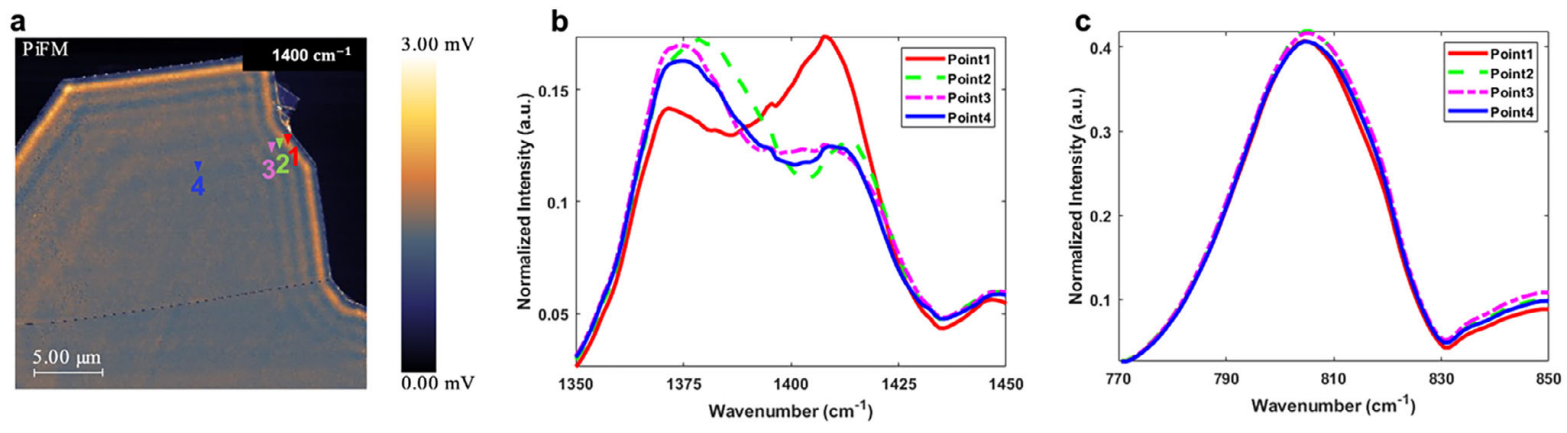
Comparison of spectra at points near the edge and further inside the sample. (a) PiFM image showing interference fringes, with spectral points marked at the fringe maxima, between fringes, and deeper inside the sample. (b) Spectra of the in‐plane phonon polariton band. (c) Spectra of the out‐of‐plane phonon polariton band.

Figure 2 examines how phonon polariton spectra evolve at a constant thickness as the polariton wave reflects from the flake edge and forms a series of interference fringes. Here, we focus exclusively on how edge‐induced interference modifies the spectral line shape of hBN phonon polaritons within a single, relatively thick flake (full thickness details shown in Figure S3).

Figure 2b shows that the in‐plane phonon polariton band contains two distinct spectral features: a lower‐frequency peak near $∼1370-1375\;cm^{-1}$ and a higher‐frequency peak near $∼1400-1410\;cm^{-1}$. At the first bright fringe, located closest to the flake edge, the higher‐frequency peak appears markedly more intense than the lower‐frequency peak. As the measurement position shifts away from the edge—at subsequent fringes and deeper into the interior—the amplitude of this higher‐frequency peak decreases, while the lower‐frequency peak becomes comparatively more prominent. Although both peaks remain present at all locations, their relative intensities vary systematically with distance from the edge.

Figure 2c presents the corresponding out‐of‐plane spectral response at the same fringe positions. Similar to the in‐plane band, the spectra exhibit more than one resonant contribution; however, unlike the in‐plane response, the overall peak shape and intensity distribution remain nearly unchanged across all measured positions. No systematic reversal of peak intensities or measurable shift in the relative contribution of individual components is observed as the distance from the edge increases.

Taken together, these measurements demonstrate that both the in‐plane and out‐of‐plane phonon polariton resonance bands contain at least two distinct spectral components that appear as a combined multipeak response. For a constant thickness, the positions of these peaks remain essentially fixed, while their relative intensities behave differently in the two bands: in the in‐plane resonance band, only the first fringe near the edge exhibits a pronounced enhancement of the higher‐frequency peak, whereas all subsequent positions across the flake show the lower‐frequency peak as the dominant feature. In contrast, the out‐of‐plane spectra retain nearly identical intensity ratios at all measured locations, indicating that their relative contributions do not vary appreciably with distance from the edge.

These observations suggest that the spectra of hBN—even at a single thickness—cannot be described by a single peak and instead arise from multiple underlying modes whose signatures are superimposed in the measured response. This motivates the need for systematic peak deconvolution and, more importantly, for extending the analysis to a range of thicknesses to determine whether the amplitudes and frequencies of these spectral components exhibit consistent trends across different layer numbers. Such a comparison provides a pathway toward identifying the physical origin of the individual peaks and understanding how each peak evolves with thickness in hBN.

### 2.3 Impact of Layer Number on Spectral Intensity and Resonance Behavior

**FIGURE 3:**
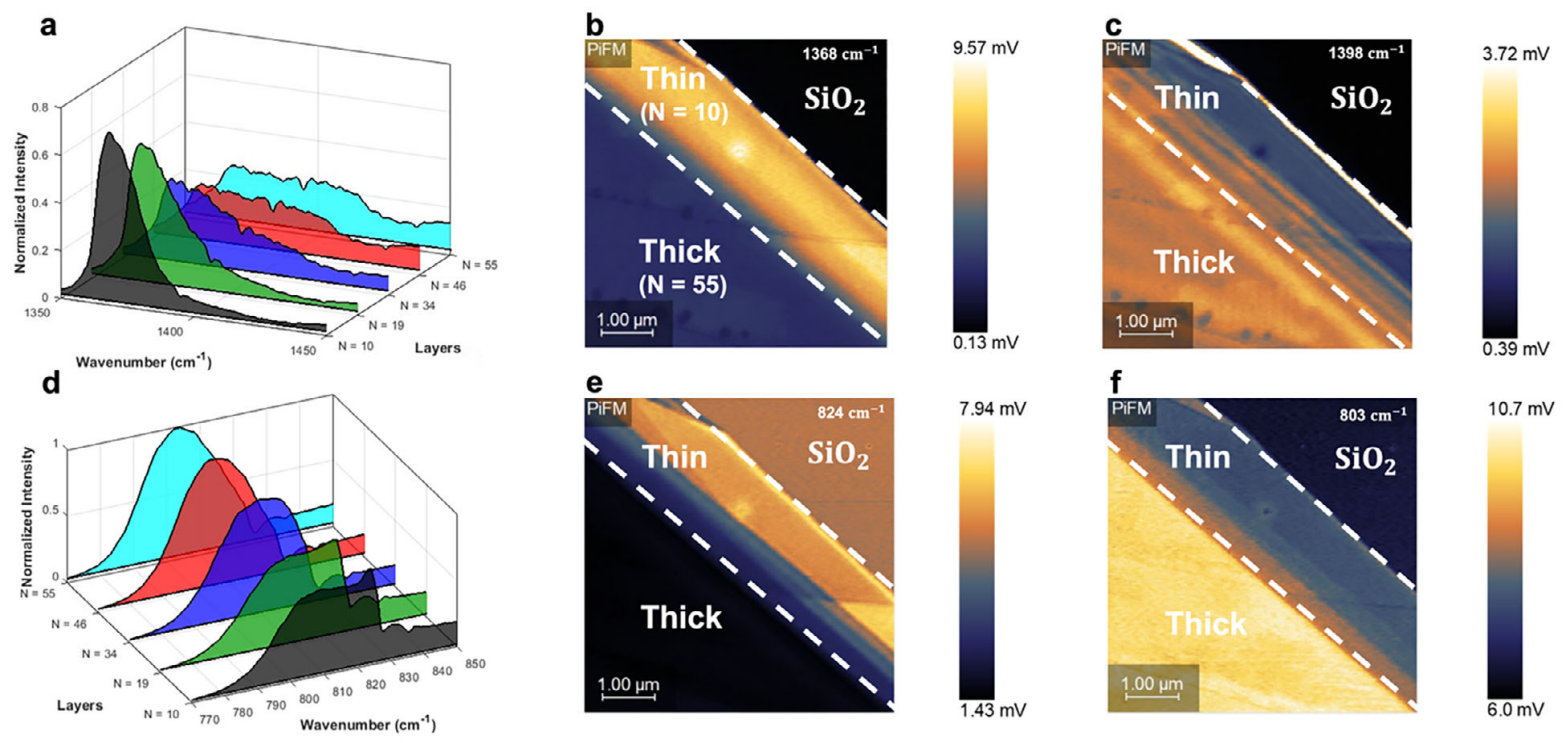
Effect of layer number variation and excitation wavenumber on phonon‐polariton responses in hBN. (a) In‐plane spectral response for varying thicknesses. Same region imaged at the resonance wavenumber of (b) the thin region (1368 cm^−1^) as well as (c) the thick region (1398 cm^−1^). (d) Out‐of‐plane spectral response for varying thicknesses. PiFM images from the out‐of‐plane resonance peak of (e) the thin region (824 cm^−1^) as well as (f) the thick region (803 cm^−1^).

To systematically investigate the relationship between hBN layer number and phonon polariton spectral characteristics, we performed correlated AFM topography and PiFM imaging on samples with spatially varying thickness (Figure S6). Figure 3 presents a comprehensive analysis of layer‐dependent optical response, demonstrating how the number of hBN layers directly influences both spectral intensity and resonance wavenumbers.

Figure 3a demonstrates the in‐plane spectral response across varying layer numbers. The spectral evolution reveals that few‐layer regions (N = 10, black curve) exhibit resonance peaks at lower wavenumbers, while many‐layer regions (N = 55, light blue curve) show shifted peaks at higher frequencies. Additionally, the spectral intensity shows a complex dependence on layer number, with thinner thicknesses often exhibiting the strongest resonance response.

In contrast, Figure 3d presents the out‐of‐plane spectral response, which exhibits the opposite trend. Few‐layer regions (N = 10, black curve) resonate at higher frequencies, while thicker regions (N = 55, light blue curve) show peaks at lower wavenumbers. The out‐of‐plane band also shows distinct intensity variations with layer number, generally exhibiting a stronger response in thicker thickness regions.

Figure 3b,c,e,f demonstrates the capability of wavenumber‐tuned imaging to selectively enhance contrast between regions of varying thickness by exploiting the layer number‐dependent shifts in phonon polariton resonance frequencies. The excitation wavenumbers for each thickness were carefully chosen based on the peak positions identified in the spectral data (Figure 3a,d), ensuring that each image captures the strongest polariton response for the corresponding layer number.

Figure 3b,c presents PiFM images acquired at the in‐plane resonance frequencies of thin (N ≈ 10) and thick (N ≈ 55) hBN regions, respectively. When the excitation frequency is tuned to match the thin region's resonance at 1368 cm^−1^, the thin areas exhibit significantly enhanced PiFM signal intensity compared to the thicker regions, creating pronounced optical contrast that clearly delineates the morphological boundaries between different layer numbers. Conversely, when imaging at 1398 cm^−1^—corresponding to the thick region's resonance—the thicker areas demonstrate stronger signal response, while the thin regions appear relatively dim. This frequency‐selective enhancement provides direct evidence of the layer‐dependent resonance frequency shifts and enables thickness‐sensitive optical mapping with high spatial resolution.

The out‐of‐plane resonance band demonstrates the opposite spectral behavior, as illustrated in Figure 3d–f. PiFM imaging at 824 and 803 cm^−1^ reveals selective enhancement of thin and thick regions, respectively, but with reversed spectral positions compared to the in‐plane band. The spectral analysis in Figure 3d (Figure S7c) shows that the thin region resonates at 824 cm^−1^ while the thick region exhibits its peak at 803 cm^−1^, demonstrating a redshift of approximately 21 cm^−1^ with increasing thickness.

These results highlight distinct thickness‐dependent behaviors for the two phonon polariton branches. The in‐plane resonance band exhibits a progressive blueshift in resonance frequency as the layer number increases, while the out‐of‐plane resonance band displays a systematic redshift with increasing thickness.

### 2.4 Quantitative Analysis of Peak Position and Spectral Broadening

In this work, the term “resonance band” refers to the overall spectral response observed within a given Reststrahlen region (RS1 or RS2), whereas “peak” denotes a local maximum extracted from the measured or deconvoluted spectra. The term “mode” refers to the corresponding underlying phononic or guided phonon‐polariton state responsible for each spectral component.

Examination of the in‐plane and out‐of‐plane spectral bands of hBN reveals that we are not dealing with a single peak for each curve, but rather complex multi‐peak structures. To achieve a better understanding of the resonance energy behavior of phonon polaritons as a function of layer number in hBN, we carried out Gaussian deconvolution analysis in which we decoupled each resonance band into two constituent peaks for both bands and investigated the behavior of these individual peaks (see Gaussian deconvolution example in Figure S9). For simplicity and to maintain consistency across all deconvolutions, we systematically divided each resonance band into exactly two peaks, as this two‐peak model consistently provided the best fit to the spectral distributions for both in‐plane and out‐of‐plane bands. This peak deconvolution analysis demonstrates that both in‐plane and out‐of‐plane resonance bands consist of multiple overlapping peaks, consistent with the presence of multiple phonon polariton modes that interact depending on the hBN layer number.

**FIGURE 4:**
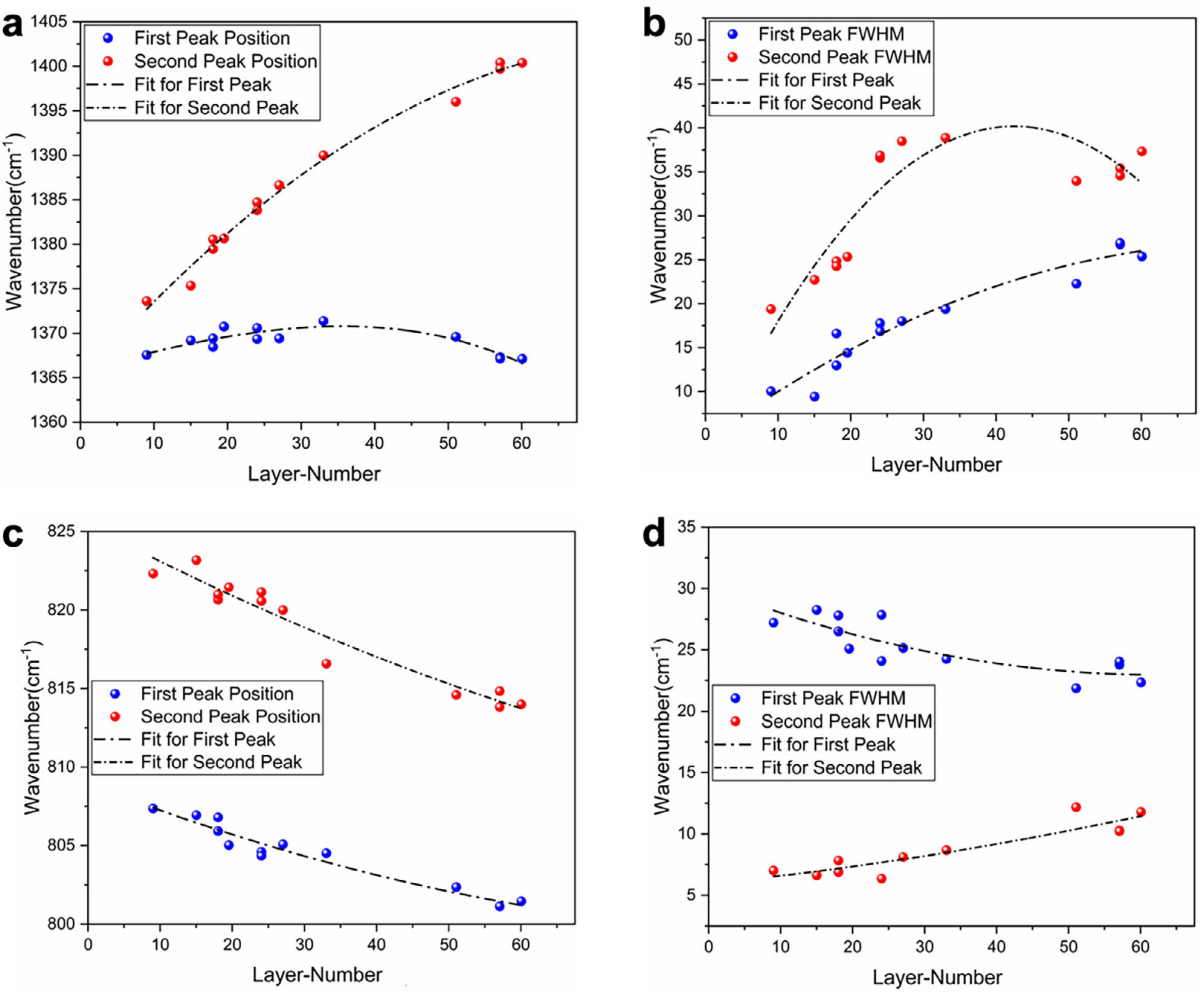
Phonon polariton resonance peak position and FWHM vs. layer number. (a) In‐plane peak position as a function of thickness. (b) FWHM of the in‐plane peak vs. thickness. (c) Out‐of‐plane peak position as a function of thickness. (d) FWHM of the out‐of‐plane peak vs. thickness.

Following the deconvolution analysis, we examined the quantitative behavior of the extracted spectral peaks as a function of hBN layer number. Figure 4a,c displays the in‐plane and out‐of‐plane fitted peak positions, respectively, plotted against layer number. The data reveal systematic and opposing trends for the two resonance bands: while the in‐plane peaks exhibit a clear blueshift with increasing thickness, the out‐of‐plane peaks demonstrate a pronounced redshift. For the in‐plane band, the lower‐frequency peak remains relatively stable around 1370 cm^−1^, while the higher‐frequency peak shifts from approximately 1375 to 1400 cm^−1^ as thickness increases from N  =  9 to N  =  60. This behavior suggests that the lower‐frequency peak corresponds primarily to the intrinsic in‐plane optical phonon response of hBN, whereas the thickness‐dependent higher‐frequency peak is associated with a guided phonon‐polariton mode. In contrast, both deconvoluted peaks in the out‐of‐plane band shift systematically toward lower frequencies with increasing thickness. Although direct dispersion mapping in this spectral region is limited by substrate overlap, the persistence of two thickness‐dependent peaks after substrate‐background correction suggests two distinct out‐of‐plane phonon‐polariton modes in the supported hBN structure.

The spectral broadening characteristics provide additional insight into the polaritonic behavior. Figure 4b,d shows the corresponding full width at half maximum (FWHM) values for both in‐plane and out‐of‐plane resonance bands as a function of layer number. The in‐plane FWHM demonstrates complex thickness dependence, with initial broadening followed by stabilization for thicker samples. The out‐of‐plane FWHM exhibits different behavior, with one deconvoluted component showing narrowing with increased thickness while the other component broadens.

Although a number of theoretical works have anticipated these layer number‐dependent trends in hBN phonon polaritons [39, 40], corresponding experimental optical validation has remained largely absent. Previous studies have primarily focused on polariton wavelength scaling with thickness (similar to what we shown in Figure 1), while systematic investigation of resonance frequency shifts has remained limited. Our findings align with recent theoretical work by Yang et al. [39], who demonstrated through numerical diagonalization and finite‐difference time‐domain (FDTD) simulations that thickness variations significantly alter the dispersion characteristics of polaritonic modes in layered materials. Similarly, Kumar et al. [40] have shown through photonic local density of states (PLDOS) calculations that thickness‐induced changes in modal confinement lead to predictable frequency shifts in hyperbolic materials. In addition, Yan et al. [41] reported curvature‐induced localized phonon polaritons in folded hBN using STEM‐EELS, while supplementary simulations in that work also indicated a layer‐dependent resonance shift. A comparison between those simulated values and our experimental results shows comparable thickness‐scaling behavior (see Figure S11).

Building on these prior studies, the contrasting behaviors of the in‐plane and out‐of‐plane spectral bands observed here arise from thickness‐dependent changes in guided phonon‐polariton dispersion within the Reststrahlen regions. For the in‐plane band, increasing thickness shifts the dominant guided mode toward higher frequencies, producing the observed blueshift. In contrast, the out‐of‐plane band exhibits redshifting peaks, consistent with different dispersion evolution arising from the anisotropic dielectric response of hBN, where the in‐plane and out‐of‐plane permittivity components govern modal confinement differently under substrate‐supported boundary conditions. To quantitatively verify this interpretation and directly relate the measured PiFM spectra to guided phonon‐polariton modes, we next compare the experimentally extracted polariton momenta with calculated dispersion curves for supported hBN flakes.

### 2.5 Thickness‐Dependent Dispersion Origin of the Spectral Resonances

To establish the physical origin of the observed thickness‐dependent spectral shifts, we analyzed guided phonon‐polariton dispersion in hBN flakes supported on SiO_2_/Si substrates using a multilayer electromagnetic model. The model was adapted from previously reported treatments of supported hBN polaritons [29], in which the p‐polarized optical response of an hBN/SiO_2_/Si stack was used to compare the calculated polariton dispersion with experimentally measured near‐field data. Details of the calculation are provided in the Supporting Information.

**FIGURE 5:**
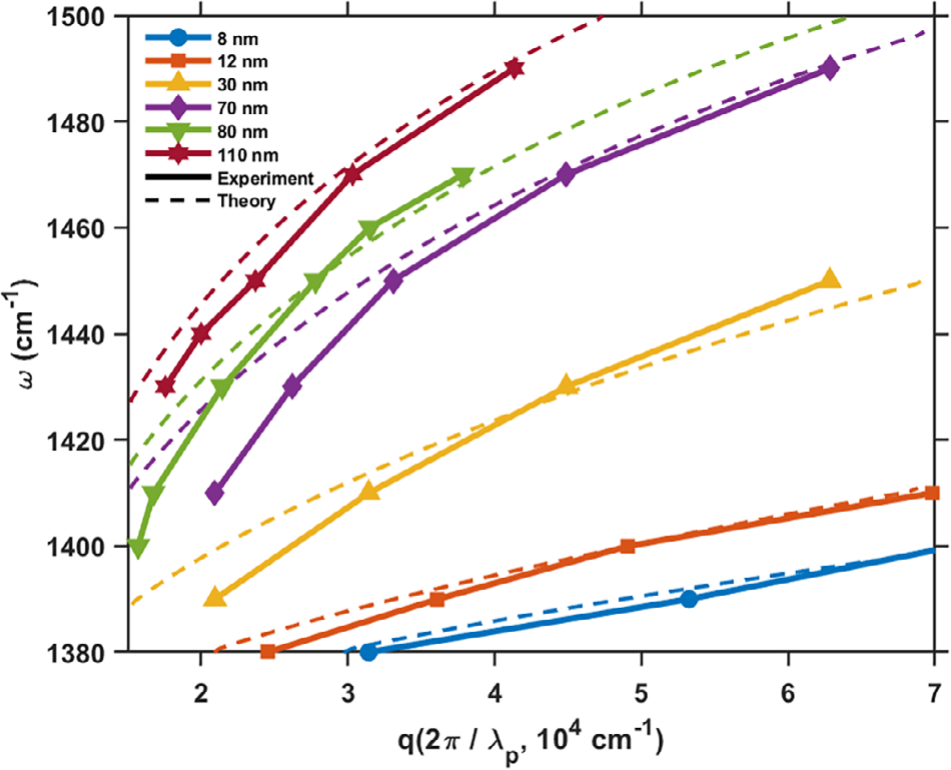
Comparison between experimentally measured phonon‐polariton dispersion (symbols, solid lines) and calculated guided‐mode dispersion (dashed lines) for hBN flakes of different thicknesses on SiO_2_/Si substrates. The investigated thicknesses of 8, 12, 30, 70, 80, and 110 nm correspond approximately to layer numbers of N ≈ 24, 36, 90, 210, 240, and 330, respectively.

Figure 5 compares the experimentally extracted phonon‐polariton momenta with the calculated dispersion curves for hBN flakes of different thicknesses. The measured data closely follow the calculated fundamental guided phonon‐polariton mode, commonly referred to as the M0 mode. This agreement indicates that the dominant PiFM response in the in‐plane Reststrahlen band originates primarily from excitation of this lowest‐order guided mode. As the hBN thickness increases, the M0 dispersion shifts toward higher frequencies at comparable momenta, reflecting the thickness‐dependent phase accumulation and electromagnetic confinement of the guided mode within the hBN slab.

This upward shift of the calculated dispersion directly explains the experimentally observed blueshift of the thickness‐dependent in‐plane peak. Because PiFM detects local photo‐induced forces generated by enhanced near fields, the spectral peak occurs when the excitation frequency efficiently couples to a supported guided phonon‐polariton mode. Therefore, as thicker flakes support the M0 mode at higher frequencies for comparable momenta, the measured in‐plane peak progressively shifts to larger wavenumbers.

The multi‐peak spectral structure observed in Figures 2 and 3 is therefore consistent with overlapping contributions from the intrinsic optical phonon response of hBN and thickness‐dependent guided phonon‐polariton modes. In the in‐plane band, the relatively stable lower‐frequency peak is attributed mainly to the intrinsic phonon response, whereas the thickness‐sensitive higher‐frequency peak follows the guided M0 phonon‐polariton mode. Their superposition gives rise to the measured two‐peak lineshape and its systematic evolution with layer number. In the out‐of‐plane band, both extracted peaks shift systematically toward lower frequencies with increasing thickness. Although the same anisotropic dispersion framework applies, direct experimental dispersion extraction in this spectral region is limited by partial overlap with the SiO_2_ substrate phonon response and the weaker near‐field coupling of out‐of‐plane phonon‐polariton modes. These limitations have been noted in prior near‐field studies of hBN polaritons [10, 27]. Nevertheless, the persistence of two thickness‐dependent peaks after substrate‐background correction suggests the coexistence of two distinct out‐of‐plane phonon‐polariton modes in the supported hBN structure, both of which evolve with thickness.

These results provide a direct bridge between momentum‐space phonon‐polariton dispersion and the spectral‐domain observables measured by PiFM. They experimentally validate theoretically predicted thickness‐tunable guided phonon‐polariton modes in hBN and reinforce the critical role of layer‐dependent dielectric anisotropy, where distinct in‐plane and out‐of‐plane permittivities govern modal confinement and dispersion differently. Our findings therefore, establish an experimental framework for engineering the spectral and directional properties of phonon‐polariton modes in layered nanophotonic systems through thickness control.

## 3 Conclusions

In this study, we present the first systematic spectroscopic investigation of layer‐dependent phonon polariton behaviors in hexagonal boron nitride using photo‐induced force spectroscopy across the intermediate thickness range of 10–60 layers. Through wavelength extraction from interference fringe patterns, we demonstrated clear linear scaling with thickness for the in‐plane band, with successful visualization of polaritonic fringes even in ultrathin samples where visibility is typically compromised. By performing wavenumber‐tuned imaging and comprehensive spectroscopic analysis across both Reststrahlen bands, we revealed distinct and opposing spectral trends: the in‐plane phonon polariton resonance exhibits a systematic blueshift with increasing layer number, while the out‐of‐plane resonance demonstrates a pronounced redshift, respectively.

Through quantitative deconvolution analysis, we find behavior consistent with theoretical predictions of layer‐number‐dependent modifications to polaritonic dispersion within the Reststrahlen band. The in‐plane band blueshift results from stronger phonon polariton dispersion at small momenta, requiring higher frequencies to maintain band structures, while the out‐of‐plane band redshift occurs as dispersion moves closer to the transverse optical phonon frequency, with enhanced dispersion at low momenta enabling access to lower frequency states.

These findings establish the experimental foundation for thickness‐engineered polaritonic devices and validate theoretical predictions of layer‐dependent dispersion control. The opposing trends observed in the in‐plane and out‐of‐plane bands show that varying the hBN thickness and thus the degree of electromagnetic confinement tunes their resonance frequencies in distinct ways. This work provides valuable insights into harnessing layer‐dependent polaritonic states for the development of advanced nanophotonic devices through precise thickness engineering in van der Waals materials.

## 4 Experimental Section/Methods

### 4.1 Sample Preparation

hBN samples were prepared using mechanical exfoliation and dry transfer methods. Bulk hBN crystals (HQ Graphene) were mechanically exfoliated using blue tape onto PDMS (Gel‐Pak X4). The exfoliated hBN flakes were transferred onto SiO_2_/Si substrate using a transfer stage (Newport XYZ‐PPP) inside a humidity‐controlled glovebox to minimize contamination and oxidation effects during the transfer process. The dry transfer technique ensured clean interfaces and preserved the intrinsic properties of the hBN samples for subsequent optical characterization.

### 4.2 Sample Characterization

Photo‐induced force microscopy (PiFM) measurements were performed using a commercial system (Molecular Vista, CA) coupled with tunable quantum cascade laser (QCL) sources. Gold‐coated silicon tips (Q:NSC15/Cr‐Au, MikroMasch) with tip radius < 35 nm were employed for non‐contact mode measurements with oscillation amplitude of 5 nm and setpoint of 75%. The scanning covered sample areas with a 512 pixel resolution at a scan speed of 15 µm/s. The QCL laser system provided tunable mid‐infrared excitation spanning 770 to 1900 cm^−1^ with a spectral resolution of 1 cm^−1^. P‐polarized laser light was focused at the tip‐sample junction, and sideband detection mode was employed to enhance surface sensitivity of the PiFM measurements for nanoscale optical characterization of the samples.

In PiFM, the detected signal originates from the optically induced tip–sample interaction force rather than far‐field scattered light. Under illumination, the confined near field at the metallic tip apex polarizes both the tip and the local sample region, generating a force‐gradient signal detected by the cantilever in sideband mode. For hBN within the Reststrahlen bands, excitation of phonon‐polariton modes enhances the local near field, thereby increasing the measured PiFM response. Consequently, spectral peaks correspond to resonant enhancement of local polaritonic fields, while fringe patterns arise from interference of propagating phonon‐polariton waves. While the PiFM response in this work is primarily interpreted in terms of photoinduced tip–sample forces arising from local polaritonic near‐field enhancement—an interpretation supported by the quantitative agreement between measured polariton momenta and calculated guided‐mode dispersion—we note that dissipative photothermal contributions may also contribute to PiFM signals under mid‐IR excitation [42, 43, 44]. In particular, local thermal expansion can modulate the tip–sample interaction and may influence weakly radiative modes such as the RS1 response [45].

For each thickness, the spectra were obtained by averaging multiple point spectra collected across the corresponding region of the flake to ensure accurate and representative measurements (see Figure S7a for full spectra). In the analysis, the in‐plane and out‐of‐plane phonon polariton bands were separated based on their distinct spectral regions. All spectra were normalized to the SiO_2_ substrate peak near 1100 cm^−1^ to account for background variations and facilitate direct comparisons across thicknesses.

A clear distinction between hBN and the substrate is observed in the in‐plane band, where the optical contrast is high, and the SiO_2_ substrate appears spectrally distinct from the hBN flake. In contrast, in the out‐of‐plane band, the optical contrast is significantly lower due to partial spectral overlap between the out‐of‐plane phonon polariton modes of hBN and the intrinsic phonon response of the SiO_2_ substrate. To isolate the hBN contribution, nearby substrate spectra were measured and subtracted from the raw RS1 spectra prior to peak analysis (see Supporting Information). This contrast difference highlights the importance of band selection for reliable thickness mapping in hBN systems (see Figure S8 for more in‐plane imaging).

Raman spectroscopy of hBN on SiO_2_/Si substrate was performed using a customized confocal Raman microscope (NanoBase XPER Raman system) at room temperature. A continuous‐wave 532 nm green laser was used as the excitation source with 1 mW incident power, and scattered light was collected using a 300 lines per mm grating for spectral analysis.

## Author Contributions

A.H. performed sample fabrication, conducted PiFM measurements, analyzed the data, and wrote the manuscript. J.S. contributed to sample fabrication. S.N. conceived and supervised the project. The manuscript was written by A.H. with input and comments from all authors.

## Conflicts of Interest

The authors declare no conflicts of interest.

## Supporting information




**Supporting File**: advs76087‐sup‐0001‐SuppMat.docx.

## Data Availability

The data that support the findings of this study are available from the corresponding author upon reasonable request.
