# Supplementary material for: Layer‐Dependent Phonon Polaritons in hBN Resolved by Photo‐Induced Force Spectroscopy
Source: Adv Sci (Weinh). 2026 Jun 12:e76087. Online ahead of print. doi: 10.1002/advs.76087 (PMC13336448; doi:10.1002/advs.76087)
Supplement: Supplementary file 1 — Supporting File: advs76087‐sup‐0001‐SuppMat.docx. [file ADVS-9999-e76087-s001.pdf]

Supporting Information

Layer-Dependent Phonon Polaritons in hBN Resolved by Photo-induced Force Spectroscopy

Amin Hajarian[1], Jiwoo Seo[1], SungWoo Nam[1,2]*

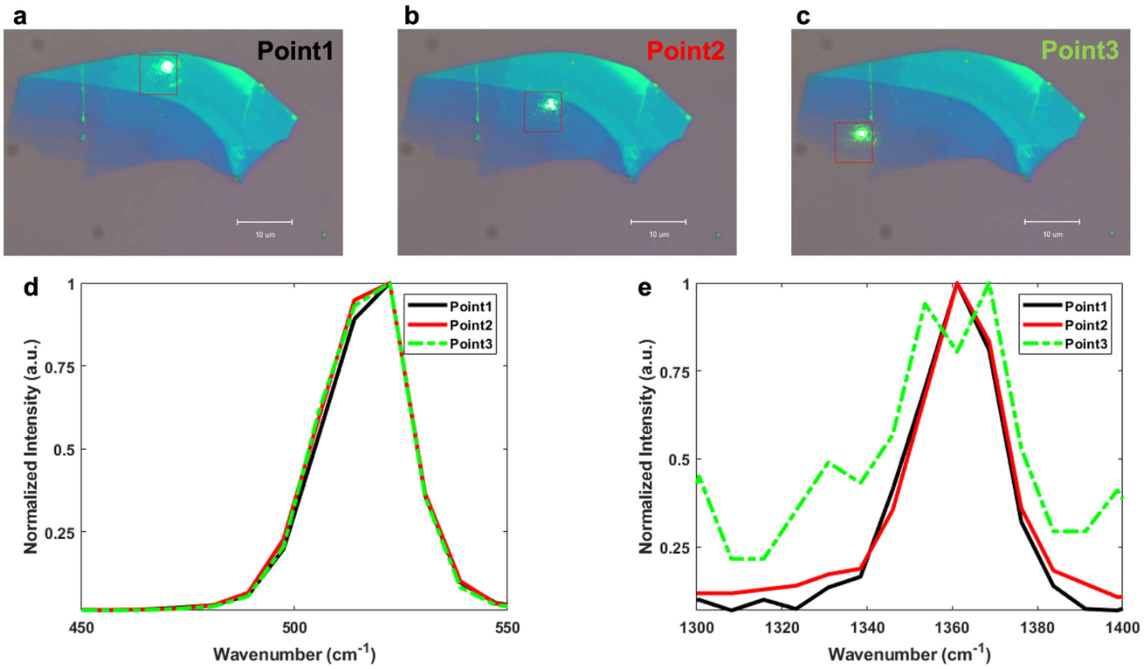

Figure S1. Effect of substrate-induced strain on the Raman signal of hBN. (a–c) Optical microscope (OM) images of the sample showing three Raman measurement points: Point 1, Point 2, and Point 3, located in regions with layer numbers N = 60, N = 40, and N = 15, respectively. (d) Normalized substrate Raman signal at the three points. (e) Normalized hBN Raman signal at the same points, illustrating the increasing influence of the substrate in thinner regions.

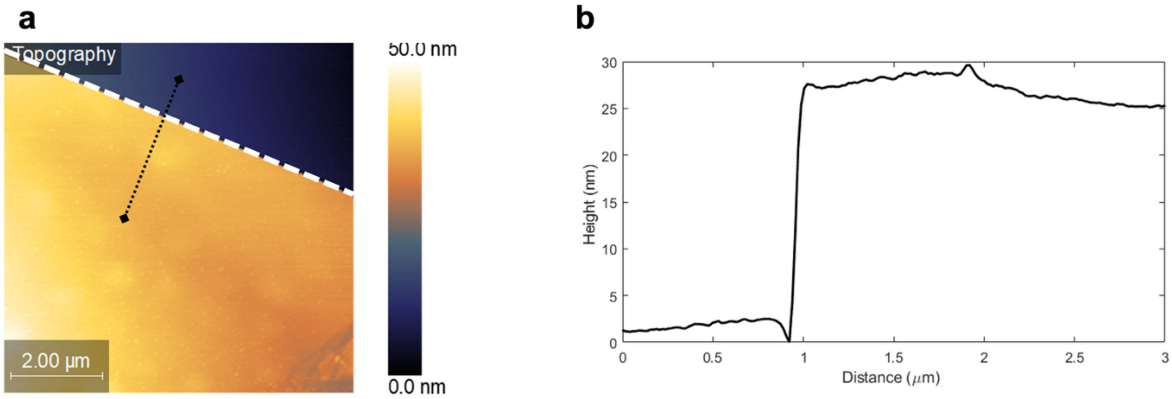

Figure S2. (a) Topography of the hBN sample, with the white dashed line indicating the sample edge. (b) Height profile along the black dashed line in (a).

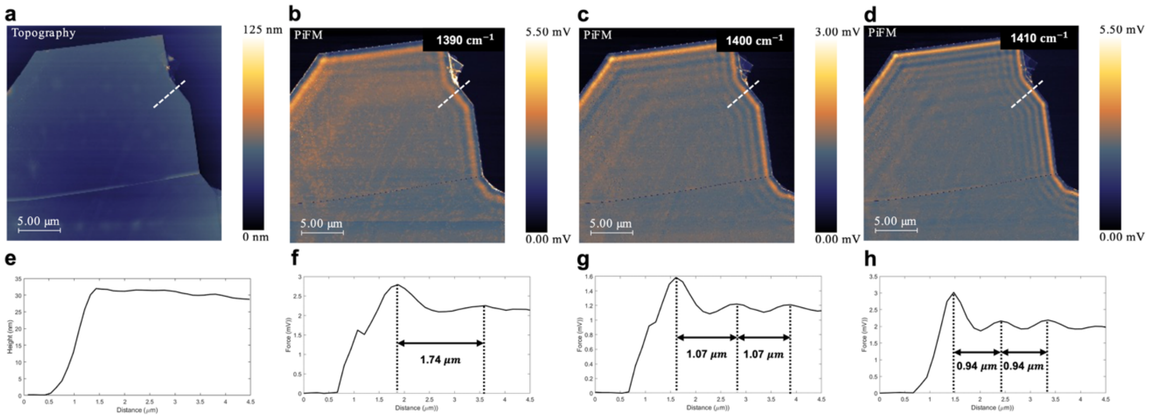

Figure S3. Change in polariton wavelength with excitation frequency. (a) Topography of the hBN sample. (b–d) PiFM images acquired at excitation frequencies of 1390 cm^{-1}, 1400 cm^{-1}, and 1410 cm^{-1}, respectively. (e) Height profile along the white dashed line in (a). (f–h) Variation in the distance between interference fringes corresponding to (b–d), demonstrating the frequency-dependent change in polariton wavelength.

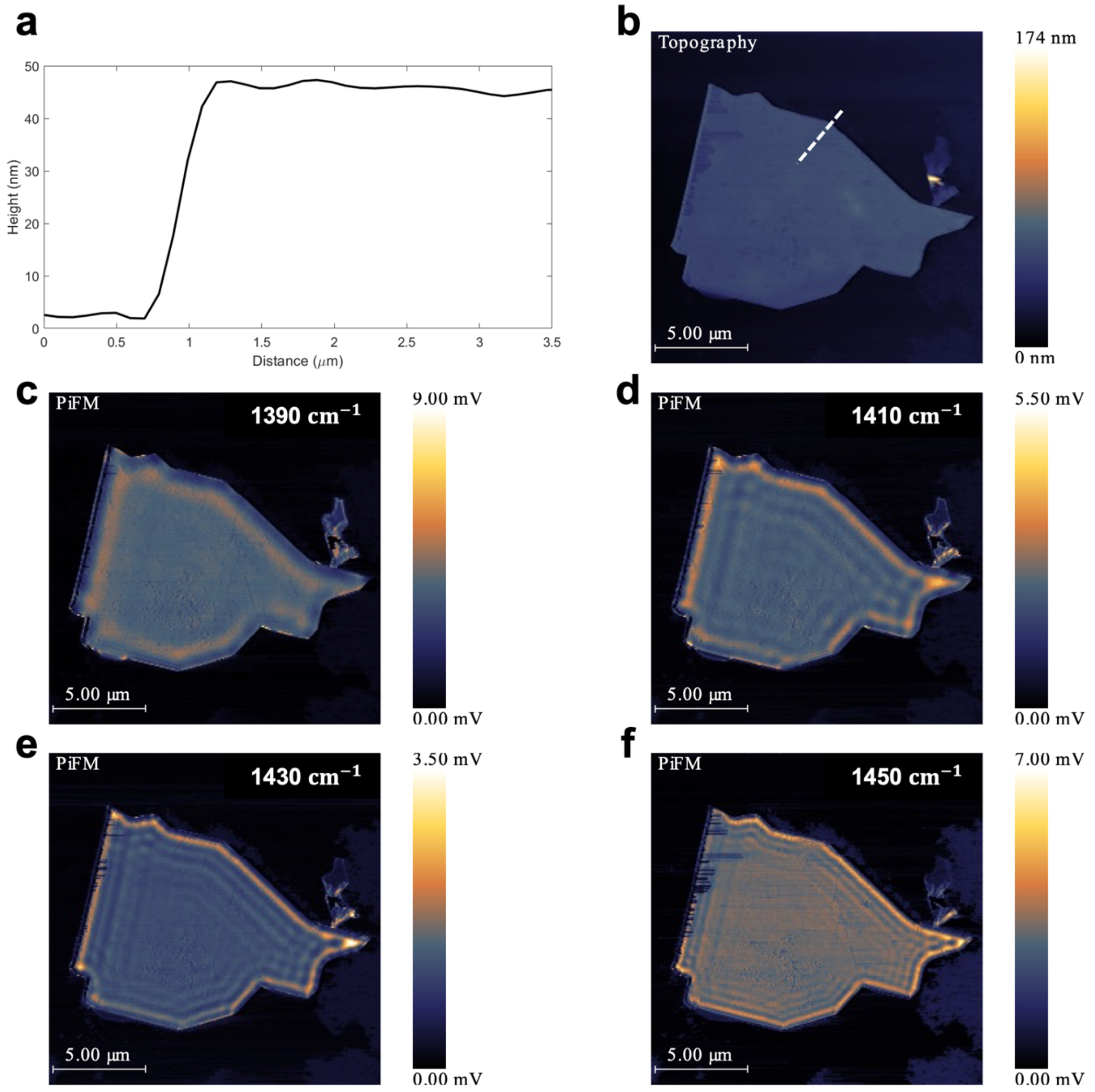

Figure S4. Change in polariton wavelength with excitation frequency. (a) Height profile along the white dashed line in (b). (b) Topography of the hBN sample. (c–f) PiFM images acquired at excitation frequencies of 1390 cm^{-1}, 1410 cm^{-1}, 1430 cm^{-1}, and 1450 cm^{-1}, respectively.

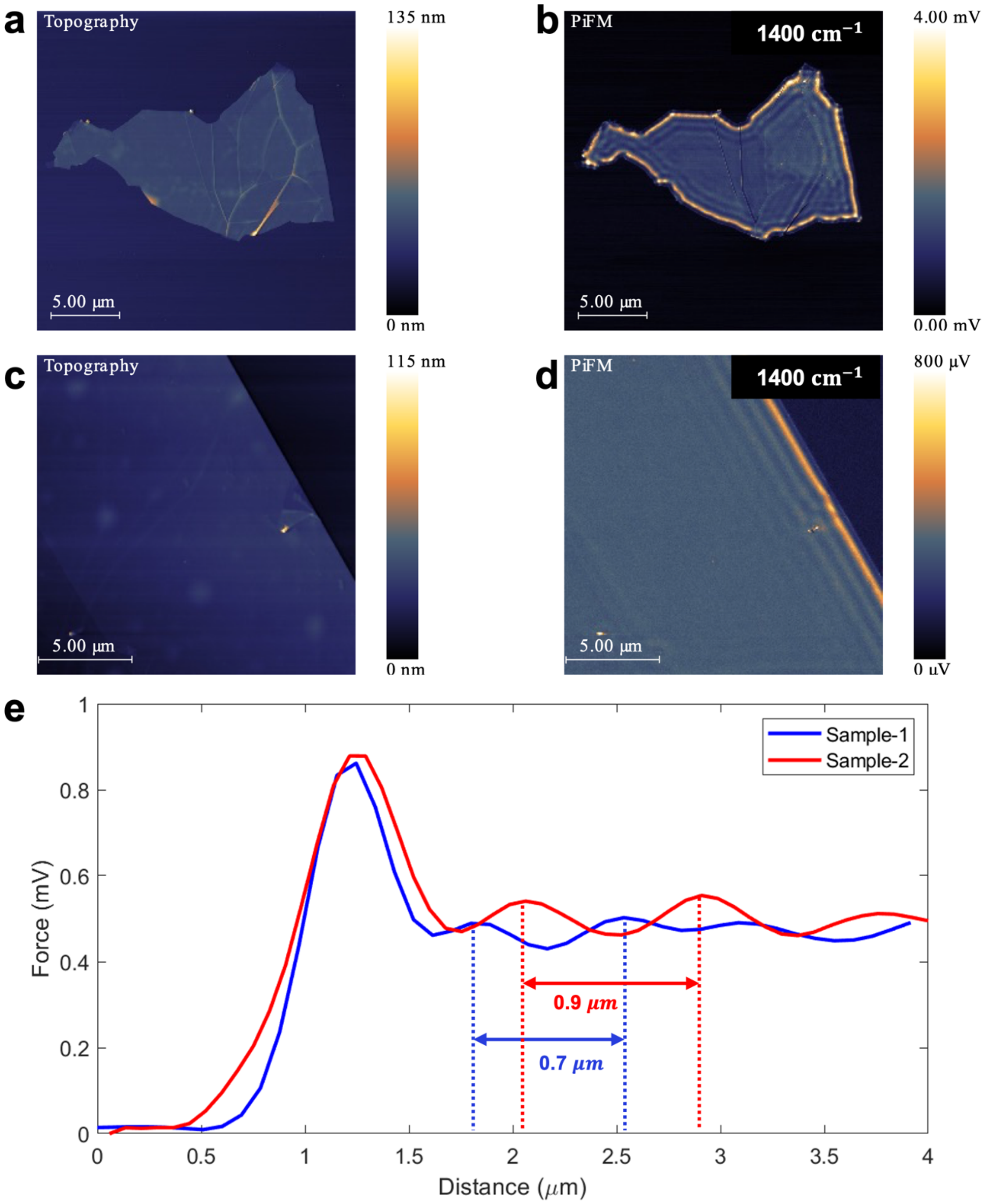

Figure S5. Change in polariton wavelength with thickness. (a,b) Topography and PiFM image of sample-1 (19 nm) acquired at excitation frequencies of 1400 cm^{-1}. (c,d) Topography and PiFM image of sample-2 (24 nm) acquired at excitation frequencies of 1400 cm^{-1}. (e) Comparison of phonon polariton wavelength variation by change of thickness at the same frequency.

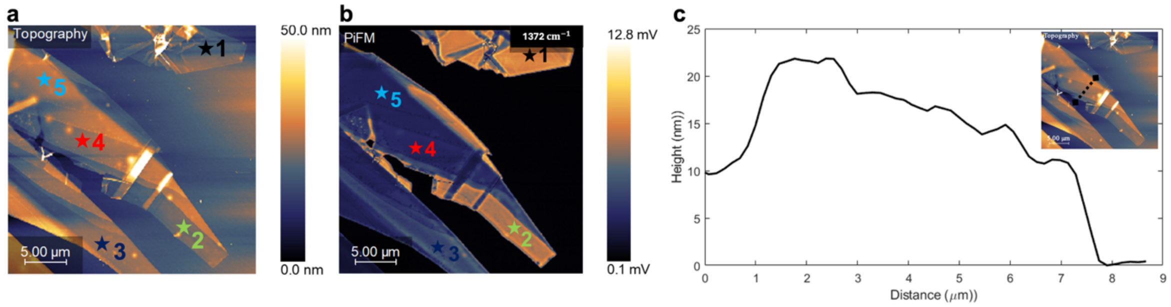

Figure S6. (a) Topography of an hBN sample on SiO_2/Si substrate, showing regions with different layer numbers (1: N = 10, 2: N = 19, 3: N = 34, 4: N = 46, 5: N = 55). (b) PiFM image (at 1372 cm^{-1}) of the corresponding topography. (c) Height profile of the step-like morphology along the black dashed line.

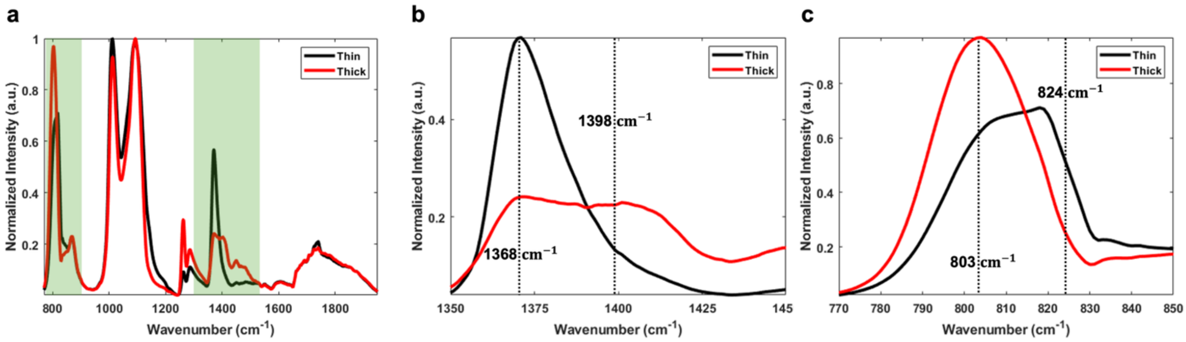

Figure S7. (a) Full spectra of the thin and thick regions. (b) In-plane spectra of the thin and thick regions. (c) Out-of-plane spectra of the thin and thick regions.

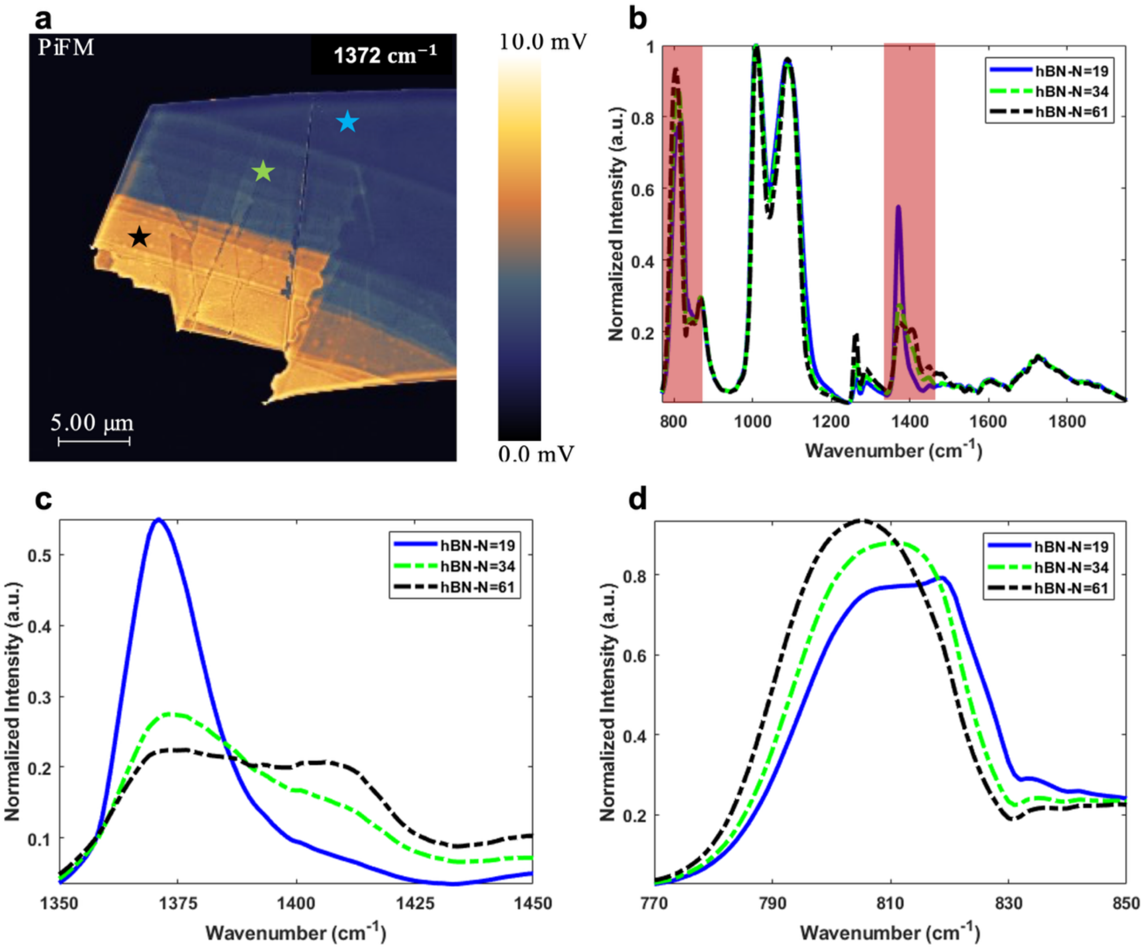

Figure S8. PiFM imaging and spectra of hBN with varying thicknesses. (a) PiFM image of hBN with different thicknesses. (b) PiFM spectra of varying thicknesses. (c) Out-of-plane spectra for different thicknesses. (d) In-plane spectra for different thicknesses.

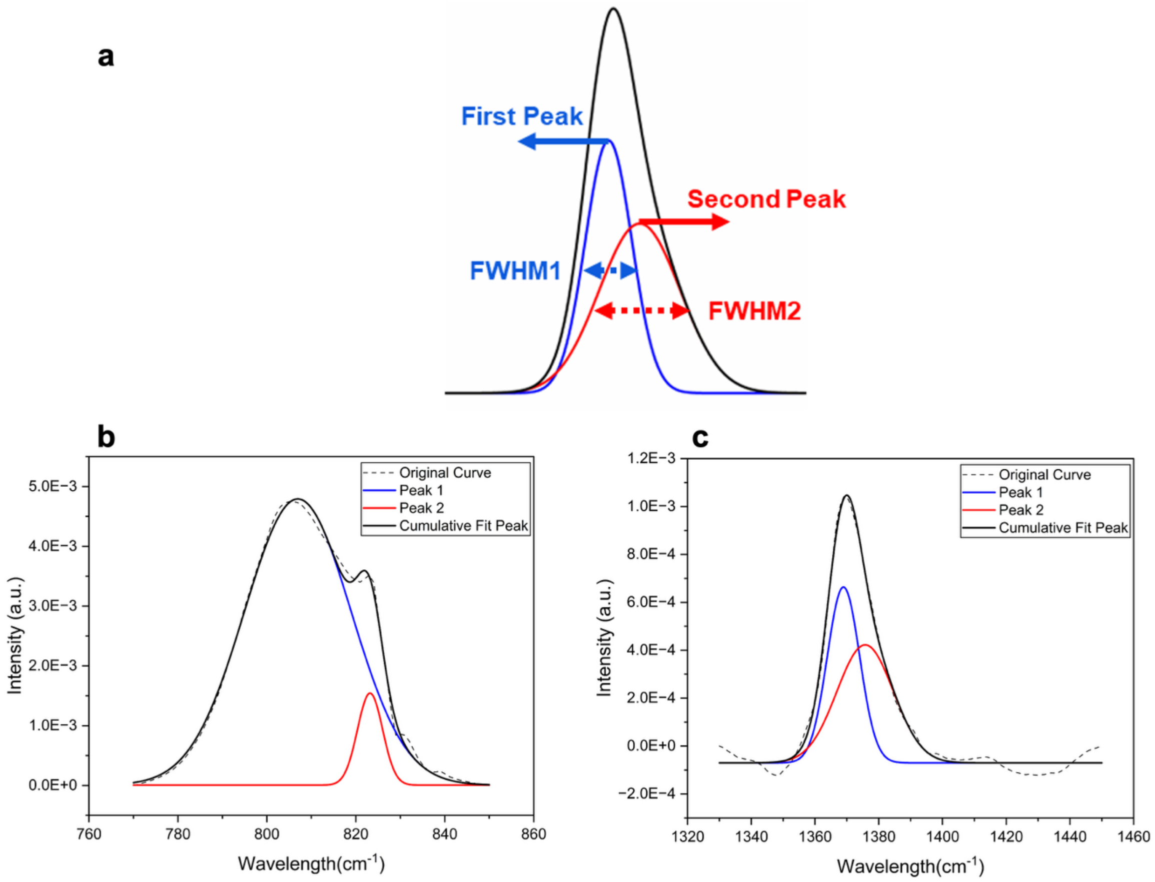

Figure S9. Gaussian curve fit decoupling. (a) Schematic showing resonance band decoupling into two components. (b) Out-of-plane resonance band decoupling for the N = 16 sample. (c) In-plane resonance band decoupling for the N = 16 sample.

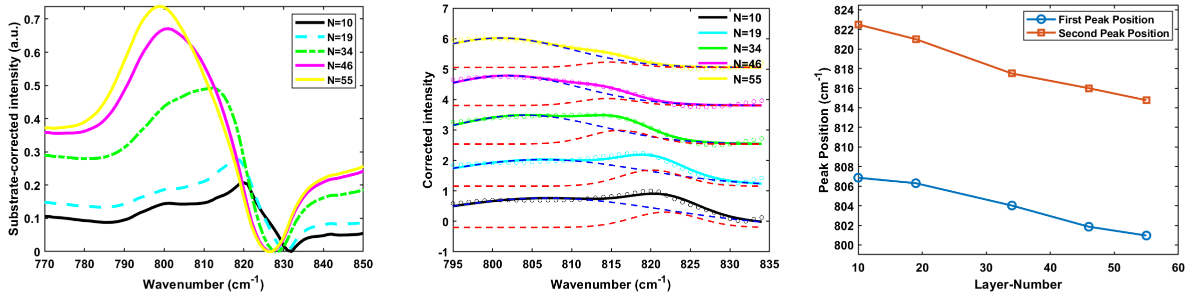

Figure S10. Removal of substrate contribution in the RS1 band. (a) Substrate-corrected PiFM spectra for hBN flakes with different layer numbers. (b) Two-peak Gaussian deconvolution of corrected spectra. (c) Extracted peak positions of the two resonance branches as a function of layer number.

Because the lower Reststrahlen band (RS1) of hBN partially overlaps the phonon-active spectral region of the SiO_2 substrate, local substrate background correction was performed prior to peak analysis.

For example, for each hBN flake thickness (N = 10, 19, 34, 46, and 55) provided in Figure 3d, PiFM spectra were collected from the hBN region and from a nearby bare substrate region under identical measurement conditions. The substrate spectrum was then subtracted from the corresponding raw hBN spectrum to isolate the hBN contribution.

Figure S10a shows the resulting substrate-corrected RS1 spectra for different layer numbers. Figure S10b presents two-peak Gaussian deconvolution of the corrected spectra. The extracted peak positions are summarized in Figure S10c.

Both deconvoluted peaks exhibit systematic redshifts with increasing layer number, confirming that the measured spectral evolution originates from thickness-dependent phonon-polariton modes in hBN. In contrast, a substrate-originated feature would be expected to remain nearly fixed in frequency.

Therefore, the reported RS1 spectral trends are attributed primarily to intrinsic hBN polaritonic behavior after removal of substrate background contributions.

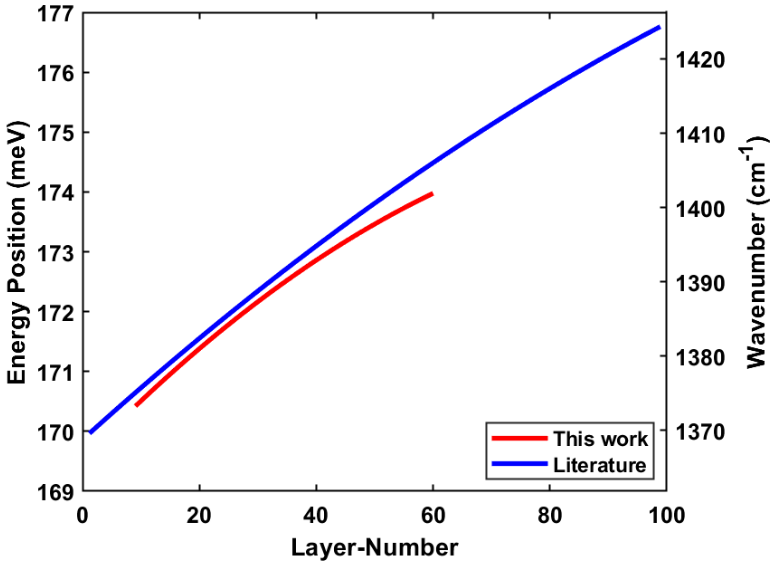

Figure S11. Comparison of the experimentally measured in-plane resonance positions in this work with the simulated layer-dependent values reported by Yan *et al.* [41]

A comparison between experimentally measured resonance energies in this work and simulated layer-number dependent phonon-polariton energies extracted from the Supporting Information of Yan *et al.* [41] is provided. Both datasets exhibit a comparable monotonic blueshift with increasing hBN thickness, supporting the thickness-dependent scaling behavior observed in the present study.

Electromagnetic model for thickness-dependent phonon-polariton dispersion

To validate the experimentally measured PiFM dispersion curves and their thickness-dependent evolution, we performed numerical calculations of guided phonon-polariton modes in hBN flakes supported on a SiO_2/Si substrate. The model was adapted from previously reported electromagnetic treatments of multilayer hBN polaritons on oxide-supported silicon substrates, particularly the framework introduced by Calandrini *et al.*, [29] in which experimentally measured polariton dispersion was quantitatively compared with calculated guided modes of supported hBN layers. Here, the calculations were used to directly correlate the measured PiFM spectral shifts with thickness-dependent phonon-polariton dispersion.

The sample was modeled as a stratified multilayer system consisting of air / hBN / 300 nm SiO_2 / Si, matching the experimental substrate geometry. Since phonon polaritons in hBN are transverse-magnetic (TM or p-polarized) guided modes, the p-polarized optical response of the multilayer structure was calculated as a function of frequency ω and in-plane momentum q.

hBN Dielectric Function

The optical response of hBN in the upper Reststrahlen band was described using a Lorentz oscillator model for the in-plane dielectric function:

$$\varepsilon_{\mathrm{hBN}}(\omega) = \varepsilon_{\infty}\left[1+\frac{\omega_{\mathrm{LO}}^2-\omega_{\mathrm{TO}}^2}{\omega_{\mathrm{TO}}^2-\omega^2-i\Gamma\omega}\right]$$

where ε_{∞} is the high-frequency dielectric constant, ω_{TO} and ω_{LO} are the transverse and longitudinal optical phonon frequencies, respectively, and Γ is the phonon damping parameter. Literature values representative of multilayer hBN were used: $\varepsilon_{\infty} = 4.90$, $\omega_{\mathrm{TO}} = 1369\ \mathrm{cm}^{-1}$, $\omega_{\mathrm{LO}} = 1621\ \mathrm{cm}^{-1}$, and $\Gamma = 18\ \mathrm{cm}^{-1}$. These parameters reproduce the negative real part of the dielectric response within the upper Reststrahlen band, enabling hyperbolic phonon-polariton propagation.

Substrate Optical Response

To more accurately describe the supported geometry, the SiO_2 layer was treated using a two-oscillator Lorentz model:

$$\varepsilon_{\mathrm{SiO_2}}(\omega) = \varepsilon_{\infty}^{\mathrm{SiO_2}} + \sum_j \frac{S_j\omega_j^2}{\omega_j^2-\omega^2-i\Gamma_j\omega}$$

while the Si substrate was described by a Drude-type dielectric function:

$$\varepsilon_{\mathrm{Si}}(\omega) = \varepsilon_{\infty}^{\mathrm{Si}} - \frac{\omega_p^2}{\omega^2 - i\Gamma_p \omega}$$

where the parameters were selected from literature values for SiO_2/Si substrates in the mid-infrared spectral range. Inclusion of the finite oxide layer is important because the dielectric environment modifies modal confinement and therefore shifts the polariton dispersion.

Multilayer Electromagnetic Formalism

For each hBN thickness d, the p-polarized Fresnel reflection coefficients at the air/hBN and hBN/substrate interfaces were calculated as:

$$r_{ij}^{p} = \frac{\varepsilon_j k_{zi} - \varepsilon_i k_{zj}}{\varepsilon_j k_{zi} + \varepsilon_i k_{zj}}$$

where k_{zi} is the out-of-plane wavevector in medium i:

$$k_{zi} = \sqrt{\varepsilon_i k_0^2 - q^2}$$

with $k_0 = 2\pi\omega$ in wavenumber units. The overall p-polarized response of the hBN slab was then written in the standard thin-film form:

$$r_p(q, \omega) = \frac{r_{\mathrm{top}} + r_{\mathrm{bottom}} e^{2ik_{z,\mathrm{hBN}} d}}{1 + r_{\mathrm{top}} r_{\mathrm{bottom}} e^{2ik_{z,\mathrm{hBN}} d}}$$

which accounts for multiple internal reflections and phase accumulation across the hBN thickness. The thickness dependence enters explicitly through the exponential propagation term $e^{2ik_{z,\mathrm{hBN}} d}$.

Extraction of Theoretical Dispersion

At each frequency, the imaginary part of the multilayer reflection coefficient, $\mathrm{Im}(r_p)$, was evaluated over the momentum range corresponding to the experimental measurements. The phonon-polariton momentum was identified as the value of q at which $\mathrm{Im}(r_p)$ reaches its maximum:

$$q_{\mathrm{PhP}}(\omega) = \arg\max_{q} \left[\mathrm{Im}(r_p(q, \omega))\right]$$

This procedure yields the dominant guided phonon-polariton branch for each thickness, which can be directly compared with the momentum extracted experimentally from PiFM fringe spacing using:

$$q = \frac{2\pi}{\lambda_p}$$

where λ_p is the phonon-polariton wavelength.

Comparison with Experiment

The calculated dispersion curves reproduced the experimentally observed thickness-dependent trend. Specifically, with increasing hBN thickness, the measured upper-band dispersion systematically shifted toward higher frequencies. Equivalently, at a fixed excitation frequency, the corresponding polariton momentum decreased with increasing thickness. The theoretical model captured this evolution across all investigated thicknesses (8, 12, 30, 70, 80, and 110 nm), confirming that the spectral shifts measured by PiFM arise from thickness-dependent guided phonon-polariton modes rather than local phonon absorption alone. These results establish a direct connection between the spectroscopic observables measured in PiFM and the known momentum-space dispersion behavior of hBN phonon polaritons, thereby providing the missing spectral-domain counterpart to previously reported near-field imaging studies.
